# Students’ Perceptions of FSBio 201, A CURE-Based Course that Scaffolds Research and Scientific Communication, Align with Learning Outcomes

**DOI:** 10.1093/icb/icab128

**Published:** 2021-06-10

**Authors:** Yee Mon Thu, Lauren B French, Bradley M Hersh, Margaret K Nelson, Lisa B Whitenack

**Affiliations:** Department of Biology, Allegheny College, 520 N. Main St., Meadville, PA 16335, USA; Department of Biology, Allegheny College, 520 N. Main St., Meadville, PA 16335, USA; Department of Biology, Allegheny College, 520 N. Main St., Meadville, PA 16335, USA; Department of Biology, Allegheny College, 520 N. Main St., Meadville, PA 16335, USA; Department of Biology, Allegheny College, 520 N. Main St., Meadville, PA 16335, USA

## Abstract

Incorporating active research opportunities into undergraduate curricula is one of the most cited elements demonstrated to improve inclusive excellence and retention in all STEM fields. Allegheny College has a long and nationally-recognized tradition of collaborative student-faculty research within the academic curriculum and as co-curricular opportunities. We present an example of the former, a Course-based Undergraduate Research Experience (CURE), FSBio 201, that has been central to Allegheny’s biology curriculum for over two decades. The course emphasizes biological research design, execution, and communication. We have coded and analyzed feedback from student evaluations and from the national CURE project database, both of which measure students’ perceptions and attitudes toward the course. The majority of the student feedback related to the course learning outcomes of fostering independent research and communication skills was positive. However, we also see areas for improvement, such as how we employ peer-to-peer mentoring and how we teach quantitative and computer-based skills. We conclude that students’ self-reported data are in line with our learning outcomes and provide FSBio 201 as a model for introducing college undergraduates to biological research.

## Introduction

A continually increasing wealth of pedagogical literature emphasizes the importance of research experiences in biology undergraduate education (Russell et al. 2007; Kuh 2008; AAAS 2011; Graham et al. 2013). The active, student-centered, and inquiry-based approach that science research exemplifies is highly effective for all students, and especially for minoritized and women students in STEM fields (Tsui 2007; Whittaker and Montgomery 2012; Wilson et al. 2012; Carpi et al. 2017; Hughes 2018). These research experiences are encouraged as independent co-curricular endeavors within and outside of the academic calendar, and as Course-based Undergraduate Research Experiences (CUREs). A wide base of literature also supports CUREs, including what defines such a course (e.g., Linn et al. 2015) and the benefits to students (e.g., Brownell and Kloser 2015; Corwin et al. 2015).

Allegheny College has been a leader in promoting undergraduate research at all levels of its curriculum for over two centuries (Coates et al. 2014). In this paper, we report students’ perception of a CURE course, Investigative Approaches in Biology (FSBio 201), that has played a vital role in the scaffolding of research experiences throughout biology students’ tenure at Allegheny since 2001, a decade before the Vision and Change publication (AAAS 2011) began the transformation of college STEM courses to include more active learning strategies. We will describe the curricular context and mechanics of the course, and then provide assessment data addressing students’ perceptions of it. Finally, we will discuss our experiences with teaching this course during the COVID-19 pandemic.

### FSBio 201 within the Allegheny curriculum

**Fig. 1:**
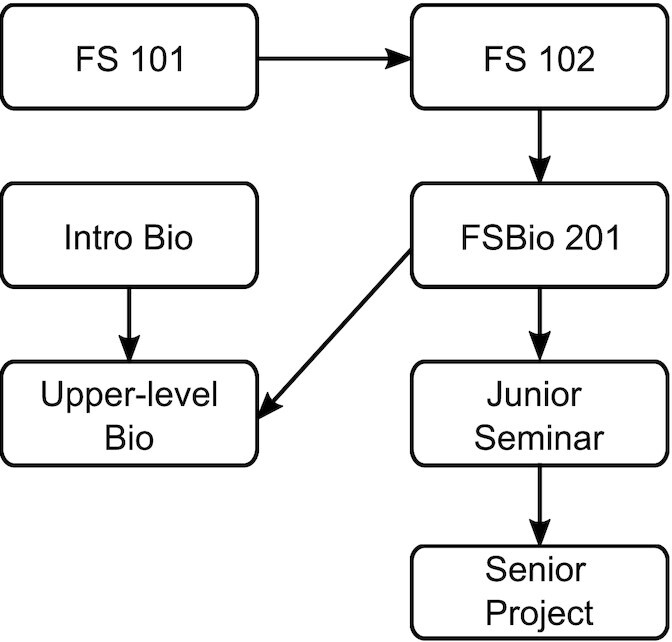
Flow chart illustrating the scaffolding of the Allegheny College curriculum. Academic writing and presentation skills in FS 101 and 102 classes prepare students for a Biology specific FSBio 201 course, which emphasizes scientific writing, oral presentation, and research skills. Students further develop these skills in junior seminar in preparation for a two-semester senior thesis project.

One of the defining features of the Allegheny curriculum is the capstone senior comprehensive project undertaken by all students, regardless of their academic background or performance. Each academic program determines the specific requirements for the capstone experience, but all students complete an original work of research, scholarship, or creative activity. To support the completion of this project, the Allegheny curriculum specifically scaffolds development of research and communication skills throughout a student’s time at the college, including FSBio 201 (Fig. 1) (Coates et al. 2014).

First-year students take two First-Year/Sophomore seminar (FS) courses, FS 101 and 102, that encourage development of oral and written communication skills. Faculty across the college develops these courses, each with a faculty-determined theme that serves as a frame for the communication exercises. At the end of these courses, students should be familiar with searching for sources; be able to analyze, summarize, and integrate information from those sources; have given formal oral presentations; and have written formal research papers that include cited references. The introduction of these skills in the first two FS courses does not, however, assume mastery.

The third FS course, FS 201 (which includes FSBio 201), is typically taken by students in their second year. Unlike FS 101 and 102, FS 201 is associated with individual programs and departments and introduces students to disciplinary conventions for speaking and writing, building on the skills introduced in the two initial FS courses and applying them toward particular content areas. Following FS 201, students enroll in a junior seminar course that further develops field-specific skills in literature search and analysis, as well as design of research and creative projects.

With this very deliberate design, the Allegheny curriculum leads students through scaffolded development of their writing, speaking, and research skills, so that all students are prepared to successfully complete their senior project. The Biology Department’s version of the FS 201 course, FSBio 201, is critical because it introduces students to the process of biological research and engages them in the experience of being an active scientist (AAAS 2011).

### FSBio 201 within the biology curriculum

While fulfilling that scaffolding role within the context of the full Allegheny curriculum, FSBio 201 is also a key requirement in the Biology Department curriculum. The introductory biology sequence at Allegheny consists of two other courses: Bio 220 and Bio 221, which generally cover introductory biology material. Importantly, neither Bio 220 nor Bio 221 have associated laboratory sections, so FSBio 201 serves as both the curricular pivot to communicating within the discipline and as the introductory laboratory course. Students must complete Bio 220 prior to FSBio 201, and many students then take Bio 221 and FSBio 201 concurrently. Pre-health students typically use FSBio 201 to fulfill their introductory biology laboratory requirement, and related programs, such as Global Health Studies, Biochemistry, Neuroscience, and Environmental Science & Sustainability, often direct students to FSBio 201 to fulfill requirements in their programs.

In FSBio 201, students begin reading articles from scientific journals, writing papers in the IMRAD (Introduction, Methods, Results, and Discussion) format, and developing their presentation abilities. Because the course is taught by a different team of instructors each semester, with a different set of experiments performed and designed by the students, we do not intend for the course to deliver a particular set of technical skills or laboratory content. To attempt to do so would result in paper-thin coverage and would present an organizational challenge to coherence, or is it clear that such an approach is necessary or productive for introductory courses (AAAS 2011). Instead, FSBio 201 prepares students for upper-level coursework and the culminating two-semester senior project through development of analytical, experimental design, and communication skills. Our student learning outcomes (LOs) for FSBio 201 state that students should be able to (1) demonstrate a general understanding of the process of biological research and scientific writing; (2) identify, interpret, and discuss relevant primary literature; and (3) present the results of independent research effectively in both written and oral formats (Table 1).

**Table 1:** Connections between the FSBio 201 learning outcomes, formative assessments, summative assessments, results from CURE survey, and results from RSE data. For CURE data, only the top five items in which Allegheny students score higher than all students are included. For RSE data, only the categories in which the number of positive comments are higher than that of the negative comments are included. CE = Course Elements, LG = Learning Gains, AS = Attitude About Science

Learning outcomes (LO)	Examples of formative assessments	Summative assessments	Results from CURE survey	Results from RSE data (positive > negative)
(1) Demonstrate a general understanding of the process of biological research and scientific writing	Reading quiz on a reading assignment on how to write a research paper Analysis of primary literature (in-class activity) Design and implement independent experiments as a group Informal group experiment proposals Drafts of individual IMRAD paper sections	Final research papers	A project entirely of student design (CE) Present results in written papers or reports (CE) Lab or project where no one knows the outcome (CE)	Designing experiments Iterative process Independence to pursue one’s own ideas Making connections to a broader picture Model Biological processes Experimental approaches
			Skills in science writing (LG) Readiness for more demanding research (LG) Ability to analyze data and other information (LG)	
			Even if I forget the facts, I’ll still be able to use thinking skills learned in science (AS) I get personal satisfaction when I solve a scientific problem by figuring it out myself (AS) The process of writing in science is helpful for understanding scientific ideas (AS)	
(2) Identify, interpret, and discuss relevant primary literature	Discussion of a primary research article with guided questions Figure analysis exercise Finding and managing papers assignment Annotated bibliography assignment Assignment on paraphrasing and citation	Final research papers and group oral presentations	Read primary scientific literature (CE)	Working with uncertainty Making connections to broader picture
			Ability to read and understand primary literature (LG) Ability to analyze data and other information (LG)	
(3) Present the results of independent research effectively in both written and oral formats	Assignment on making figures Drafts of individual IMRAD paper sections Preliminary data presentations	Final research papers and group oral presentations	Present results orally (CE)	Writing Oral communication
			Skill in how to give an effective oral presentation (LG)	
			Explaining science ideas to others has helped me understand the ideas better (AS)	

Research and communication skills are further engaged by the junior seminar courses, which examine specific subdisciplines of biological research. Each junior seminar, however, is intended to build literature analysis, research design, and writing skills, regardless of specific biological sub-discipline. The biology junior seminar culminates in the written and oral presentation of a research proposal. For many students, this proposal serves as the basis for their senior project.

## Course organization and mechanics

FSBio 201 consists of two or three multi-week project modules designed to illustrate investigative approaches at different levels of biological organization: molecular/cellular, organismal/physiology, and population/ecosystem (Table 2, Supplement 1). This organization mirrors that of our upper-level courses and is amenable to a wide range of biological systems and experimental approaches. When determining the departmental schedule each year, the chair of the department takes care to ensure faculty of diverse areas and/or subfields are represented within each FSBio 201 course offering.

**Table 2:** A list of modules that had been taught in FSBio 201. The topics cover three broad areas of biology: molecular/cellular, organismal/physiology, and population/ecosystem

Module topic
Plant peroxidase isoenzyme analysis
UV mutagenesis and repair in *Serratia marcesens*
*Dictyostelium discoideum* chemotaxis
Dog SNP genotyping
Using DNA barcodes to assess insect and plant diversity
Genes and environment interaction (*Saccharomyces cerevisiae*)
Cancer cell biology
Teratogens and zebrafish development
Bean beetle (*Callosobruchus maculatus*) microbiomes
Crayfish muscle physiology
Human dive response
Shark tooth biomechanics
Salamander jumping kinematics
Mating behavior and maternal care in the ring-legged earwig (*Euborellia annulipes*)
Physiological ecology of plethodontid salamanders
Goldenrod gall ecology
Phytoplankton community structure
Epidemiology

The course consists of two 3-h meetings per week. In the two-module system, students spend about 7 weeks in each module, while in the three-module system, students spend about 4 weeks in each module. Both systems employ previous FSBio 201 students as peer teaching assistants (TAs). The TAs assist with laboratory activities and provide students feedback on both oral presentations and rough drafts of lab reports.

The organization of the three-module system follows a weekly pattern. In the first week of the module, the general subject matter and the laboratory techniques of the module are introduced. The students (in groups of 3–4, with a total of 3–5 groups in each classroom) conduct an instructor-designed experiment so they can practice the laboratory techniques. The second and third weeks are devoted to independent experiments, which student groups develop based on some combination of *a priori* knowledge from other courses, reading scientific literature, or moments of discovery and/or failure during in-class experiments. In both weeks, the first session is devoted to informal student presentations, in which the students describe the results of the previous week’s experiment and discuss their research and plans for the independent experiment. The second session in each of the second and third weeks is used for the students to conduct their independent group experiments. The final week of the module is devoted to preparing for and presenting the groups’ findings in formal oral and written formats. In the three-module system, the major assignments consist of three formal lab reports and three formal oral group presentations. Minor assignments can include a syllabus exercise, graphing tutorial, and literature search tutorial (Table 1). Participation in the form of regular engagement in class activities, group work, and informal presentations is also part of the final grade.

In the two-module system, the major assignments consist of two formal lab reports and two formal oral group presentations with a variety of activities and smaller assignments. Both systems share the investigative experimental approach in which the students design and test their own research questions in small groups based on a preparation they learn and practice in class. Fewer modules in the semester results in more time per module (7 weeks vs. 4 weeks), allowing instructors to use the additional class meetings to include additional formative assessments and activities to further explore the mechanics of how to locate, read, interpret, and write scientific papers. These activities include guided figure analysis, guided reading of paper sections, and abstract writing.

## How do students perceive FSBio 201?

The establishment of FSBio 201 in 2001 predates the conception of “backwards course design” and thus the course is not explicitly structured based on this model. This is the first retrospective study to formally assess students’ attitudes toward this course. We had two sources of data available for this purpose: Classroom Undergraduate Research Experience (CURE) survey responses and end-of-semester evaluations (Reports of Student Experience, RSEs) from Allegheny College. **The goal of these data analyses is to evaluate how well students’ perceptions of FSBio 201 align with the learning outcomes**.

### CURE survey

To understand the effects of FSBio 201 on students’ perceived skills and attitude toward science, we administered the Classroom Undergraduate Research Experience (CURE) survey—an assessment tool developed by a collaboration of faculty members from Grinnell, Hope, Harvey Mudd, and Wellesley Colleges—over multiple semesters. Briefly, the CURE survey collected data on student demographics, intention to declare a major in science, post-graduation plans, and reasons for taking the course. In addition, the survey evaluated students’ perceptions of the course within a cohort of students, compared to all CURE participants, using a Likert scale (ranging from 1 to 5) in three broad categories—Course Elements (1 = no or very small gain, 5 = very large gain), Learning Gains (1 = smallest gain, 5 = largest gain), and Attitudes about Science (1 = strongly disagree, 5 = strongly agree)—with multiple questions/items in each category. For Course Elements and Attitudes about Science, pre- and post-course scores were collected. For Learning Gains, the scores indicate the self-reported perceived improvement as a result of completing the course, and so this category only appeared in the post-course survey. Additional information about the survey can be found at the CURE survey website (https://sure.sites.grinnell.edu/cure-survey/).

**Fig. 2:**
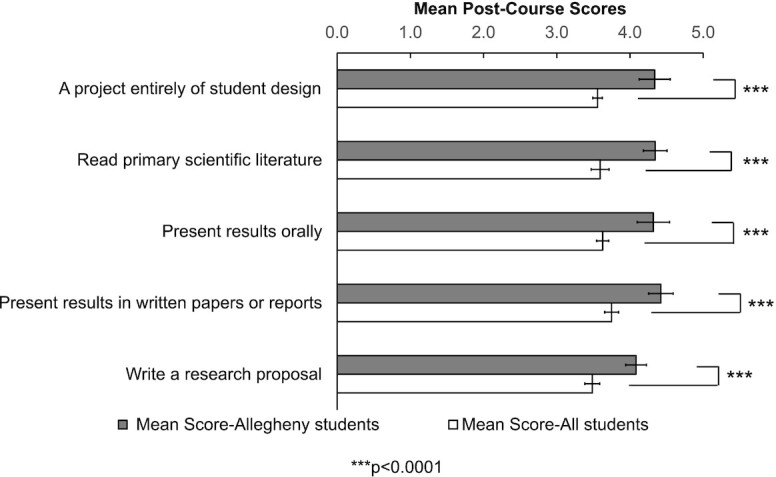
Mean post-course scores of Course Elements from the CURE survey. Only the top five items where Allegheny students score higher than all students are shown (1 = no or very small gain, 5 = very large gain). From Spring 2014 to Spring 2017, 210 Allegheny students and 41,659 students in total (including the 210 Allegheny students) completed the post-course survey. Unpaired *t*-tests were performed to determine statistical significance (full results can be found in Supplement 2, *n* = 9 for Allegheny students and *n* = 5 for all students, df = 12). Error bars represent standard deviations.

**Fig. 3:**
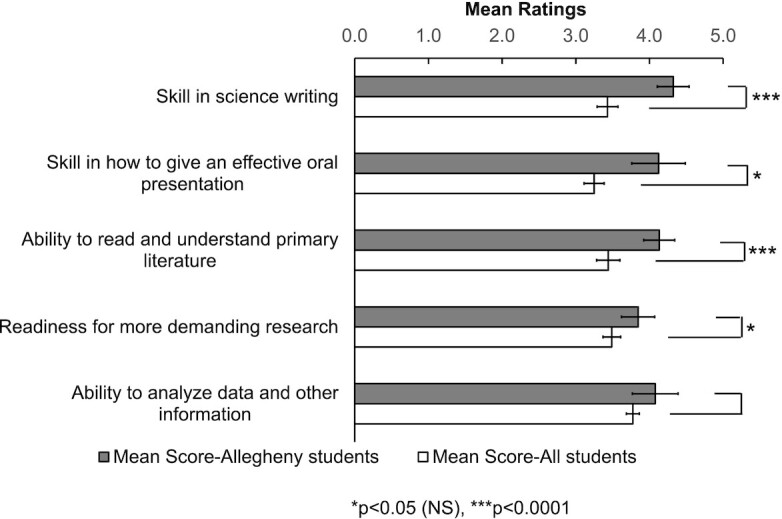
Mean ratings of Learning Gains from the CURE survey. Only the top five items where Allegheny students score higher than all students are shown (1 = smallest gain, 5 = largest gain). From Spring 2014 to Spring 2017, 210 Allegheny students and 41,659 students in total (including the 210 Allegheny students) completed the post-course survey. Unpaired *t*-tests were performed to determine statistical significance (full results can be found in Supplement 2, *n* = 9 for Allegheny students and *n* = 5 for all students, df = 12). Error bars represent standard deviations, NS = non-significant with Holm’s sequential Bonferroni method.

**Fig. 4:**
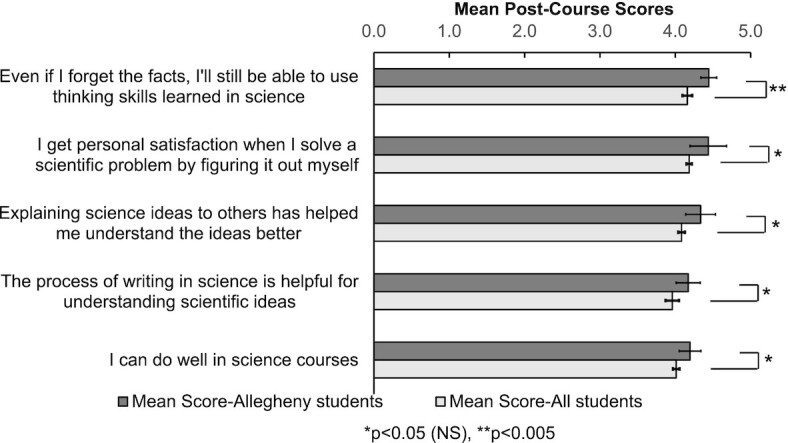
Mean post-course scores of Attitudes about Science from the CURE survey. Only the top five items where Allegheny students score higher than all students are shown (1 = strongly disagree, 5 = strongly agree). From Spring 2014 to Spring 2017, 210 Allegheny students and 41,659 students in total (including the 210 Allegheny students) completed the post-course survey. Unpaired *t*-tests were performed to determine statistical significance (full results can be found in Supplement 2, *n* = 9 for Allegheny students and *n* = 5 for all students, df = 12). Error bars represent standard deviations, NS = non-significant with Holm’s sequential Bonferroni method.

The CURE survey was administered pre- and post-course across multiple semesters from 2014 to 2017. In addition, the CURE project compiled data from all survey administration sites, allowing us to compare Allegheny FSBio 201 students to an “All-Student” survey group. For each question in the CURE survey, we considered the class mean of the Likert scale rating from one section of FSBio 201, matched with the mean from All Students for that particular academic year (which is how the All Students data are reported), as an independent data point. For Course Elements and Attitudes about Science, only post-course scores were used. Scores from FSBio 201 were compared to those from all students who had taken the survey ( = “All Student”). We evaluated data for all questions in each category, followed by statistical analyses (described below). In Figs. 2 –4, we report only the “top five” items: those items with the largest mean score differences between Allegheny students and All Students with Allegheny students scoring higher than All Students. Our full data can be found in Supplement 2.

In total, 210 Allegheny students completed the post-course survey from Spring 2014 to Spring 2017, compared to 41,659 students in total (including the 210 Allegheny students). The Allegheny student data could not be deconvoluted from the All Student data, as both data sets were provided as means from the survey administrators. Given the difference in magnitude between the sample sizes for Allegheny students versus All Students, we assumed that the data from Allegheny students would have negligible effects on the mean responses for All Students. Based on this assumption, we ignored the lack of independence and compared mean responses using unpaired *t*-tests. Statistical analyses were performed in GraphPad, with ɑ = 0.05 and significance levels adjusted with Holm’s sequential Bonferroni method for all tests. Results for all *t*-tests can be found in Supplement 2.

#### Top five items in the CURE survey demonstrate that students perceived FSBio 201 as a course that prepares them for scientific process and communication

To determine how well students’ perception aligns with the LOs of FSBio 201, we first evaluated the top five items under each broad category. Overall, FSBio 201 students reported that they benefited most in science communication skills. Furthermore, they also reported gaining experience in designing experiments and understanding primary literature. Under Course Elements, the top five questions with a significantly higher self-reported gain for our students than for All Students pertain to unscripted lab projects driven by students, reading primary literature, and communicating research findings (*P* < 0.0001) (Fig. 2, Supplement 2). Similarly, under Learning Gains, self-reported scores in science communication and approaching primary literature show the largest differences, with Allegheny students scoring significantly higher than all CURE participants (*P* ≤ 0.0003) (Fig. 3, Supplement 2). The self-reported gain in “readiness for more demanding research” and “ability to analyze data and other information” are also higher compared to all students, although the differences are not statistically significant once adjusted with Holm’s sequential Bonferroni method (Fig. 3). Under Attitudes About Science, only one item is statistically significant (“Even if I forget the facts, I’ll still be able to use thinking skills learned in science”) (*P* = 0.0010) (Fig. 4, Supplement 2). Nonetheless, we are encouraged to observe that, like the previous categories, two items related to science communication (“explaining science ideas to others has helped me understand the ideas better” and “the process of writing in science is helpful for understanding scientific ideas”) are among the top five (Fig. 4). We were also pleased to see “I can do well in science courses” included in the top five, as self-efficacy plays a role in STEM performance and perseverance (Bandura and Locke 2003; Bong and Skaalvik 2003; Rittmayer and Beyer 2008; Sithole et al. 2017). Overall, students’ self-assessment through the CURE survey nicely align with learning outcomes of FSBio 201, especially LO 1 and 3 (Table 1).

#### Bottom five items in the CURE survey demonstrate that students’ perception of FSBio 201 aligns well with the course learning outcomes

The bottom five scoring items (those items with the largest mean score differences between Allegheny students and All Students with Allegheny students scoring lower than All Students) also inform us about how students perceive the FSBio 201 learning outcomes (Supplement 2). Under Course Elements, the bottom five items include test-taking, keeping lab notebooks, reading a textbook, working on problem sets, and presenting posters. Allegheny students rated themselves statistically lower than All Students for test-taking and working on problem sets (*P* ≤ 0.0008) (Supplement 2). Because the FSBio 201 course does not emphasize any of these Course Elements, these data suggest that the positive alignments observed between the CURE data and our learning outcomes for our top five items also reliably reflect true self-reported gains for our students. Under Learning Gains, our students’ mean self-reported score is lower than that of all students for “clarification of a career path” (*P* = 0.0024), which is not widely discussed in the course (Supplement 2). For the other four items in the bottom five, the differences between Allegheny students and All Students are not statistically different. Under Attitudes About Science, the questions associated with categories that fall into the bottom five are phrased such that a *lower* score (disagreement) indicates students’ perception of the scientific process is closer to its true nature. Allegheny students scored significantly lower than All Students in most categories (*P* < 0.003 except for “I wish science instructors would just tell us what we need to know so we can learn it,” which has *P* = 0.0036) (Supplement 2). These self-reported responses are indicative of a realistic understanding of the nature of science and its relationship to other fields. For example, FSBio 201 students disagreed more with the statement “science is not connected to non-science fields such as history, literature, economics, or art.” Although this statement is not directly related to the learning outcomes of FSBio 201, the major/minor requirement of Allegheny College, for which students must choose a minor in a different academic division (i.e., Humanities, Natural Sciences, Social Sciences) than their major, may equip FSBio 201 students to make connections to broader fields more readily.

### End-of-semester evaluations (RSEs)

Like most colleges and universities, Allegheny College employs surveys (RSEs) at the end of each semester for students to provide feedback to their instructors. We analyzed RSEs from the Fall 2013 through Fall 2019 semesters, using data from two different versions of the RSE form. A description of these forms can be found in Supplement 3.

The variables we coded fall into three broad categories: (1) content specific to a module (organisms/experimental subjects, biological processes/sub-disciplines, and experimental approach/techniques specific to a sub-discipline); (2) different facets of research universal to all sub-disciplines (designing experiments, iterative process, independence, working with uncertainty or recognizing the aspect of discovery, collaboration, and making connections to a broader picture); and (3) communication skills (oral presentation and writing a research paper) (Table 3). Measuring these variables allows us to assess how closely students’ perceptions and the learning outcomes of FSBio 201 align. In addition, we included a category to indicate students’ attitudes toward peer-to-peer learning from teaching assistants and two categories that assessed students’ overall response to the module or course (desire to further student’s interest and general likes/dislikes). We used the same coding scheme for each category (positive, negative, neutral). To minimize subjectivity, the authors did not code RSEs from their own modules. Only one coder was responsible for a given semester’s set of RSEs. Due to this limitation, we did not perform any statistical analyses. We coded RSEs from 19 consecutive semesters (Fall 2013–Fall 2019) that encompassed 48 total modules and 1551 individual comments. A detailed description of the coding scheme and the coding guidelines along with examples can be found in Supplements 3 and 4, respectively. Protocols for this part of the study were approved by the Allegheny College Institutional Review Board (protocol 2020–05).

**Table 3:** Different categories used for coding RSEs. Some categories are module-specific whereas, others pertain to the general process of science. Categories related to communication skills, peer-to-peer teaching, and two additional categories were also used

Overview of the coding guidelines	
Contents specific to a module	
Use of model organisms/experimental subjects	
Study of biological processes and sub-disciplines	
Experimental approaches or techniques specific to a sub-discipline	
Different facets of research universal to all sub-disciplines	
Designing experiments	
Iterative process	
Independence to pursue one’s own ideas	
Working with uncertainty or recognizing the aspect of discovery	
Collaboration	
Making connections to a broader picture	
Communication skills	
Writing a research paper	
Oral presentation	
Others	
Peer-to-peer learning	
Desire to further student’s interest	
General likes/dislikes	

#### RSE data generally show positive perceptions of the course in a majority of the surveyed categories

**Fig. 5:**
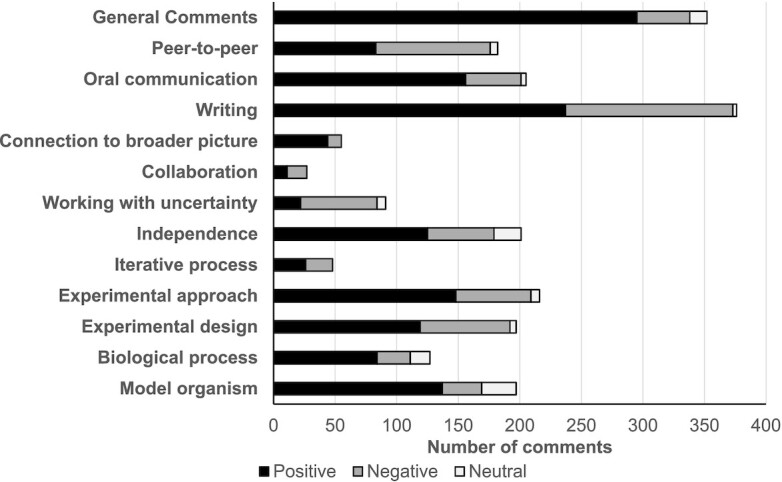
Distribution of RSE comments (positive, negative, or neutral) across different categories. The data represent coding of RSEs from 19 consecutive semesters (Fall 2013–Fall 2019) that encompassed 48 total modules and 1551 individual comments.

Because the RSE prompts are open-ended and are not explicitly limited to our coding categories, we identified a large number of comments that did not fit our coding scheme (37.0% of total responses) (Table 4). These comments included personal attributes of the instructor and comments on speed of the return of work. One theme that emerged from the “other” category was the importance of instructor enthusiasm and energy. Instructor enthusiasm can motivate students (Alsharif 2011), and the RSE data suggest conveying passion for science may positively change the perception of FSBio 201. In general, student comments on the RSEs demonstrate a positive attitude toward FSBio 201. Ten out of 13 categories had more positive comments than negative ones, ranging from 54% (iterative process) to 84% positive (general like/dislike of the course or module) (Fig. 5).

**Table 4:** Percentage of RSE comments for each category. Categories are arranged in a descending order of percentages

Summary of RSE data	
Other comments	37.01%
Writing a research paper	24.24%
General likes/dislikes	22.7%
Experimental approaches or techniques specific to a sub-discipline	13.93%
Oral presentation	13.22%
Independence to pursue one’s own ideas	12.96%
Use of model organisms/experimental subjects	12.7%
Designing experiments	12.7%
Peer-to-peer learning	11.73%
Study of biological processes and sub-disciplines	8.19%
Working with uncertainty or recognizing the aspect of discovery	5.87%
Making connections to a broader picture	3.55%
Iterative process	3.09%
Collaboration	1.74%
Desire to further student’s interest	1.16%

#### Through students’ self-reported RSE data on content specific to a module, we infer that students gain a general understanding of biological research (LO 1)

To gain a better understanding of student attitudes about particular modules and subfields, we more closely examined three categories: “model organism,” “biological process,” and “experimental approach.” When we examined mentions of specific model organisms, no broad taxonomic group had more negative than positive comments (Supplement 5). This result held true whether mentions of humans were categorized separately or with other animals. Only 8.2% of comments fell under “biological process,” and comments were largely positive (66%). We could not find a specific trend under “biological process” as students used a wide range of vocabularies to describe the same biological process. Self-reported data on another module-specific category, “experimental approach,” reveal sub-discipline specific practices that students enjoy or do not enjoy. These data also suggest that students are gaining a more realistic view of how science is done. For example, total responses about fieldwork were 26.4% of all “experimental approach” responses, and 92.9% of those were positive. Biology programs, including our own, sometimes perceive that a high percentage of biology majors are pre-health students and that they are interested only in disease-related research. Students’ reported appreciation for fieldwork suggests that these perceptions are not accurate. On the other hand, two aspects within “experimental approach” that generated negative perceptions were (i) coming in outside of class time (92.9% negative) and (ii) having to wait for procedures to run (90.9% negative). Working scientists understand the inevitability of these kinds of inconveniences (when has a biologist *ever* made good on their promise to only be stopping into the lab for 5 min?), and it is not surprising that students might have a negative reaction to their initial exposure to this scientific reality. Overall, data in these categories provide us the metrics to gauge students’ perception of the process of science (LO 1) not only as a way of approaching a problem but also as an undertaking in which specific common practices are followed to achieve a successful outcome (Table 1). These realistic experiences are, we hope, informative for students who are considering a career in science.

#### RSE data on scientific process (different facets of research universal to all sub-disciplines in Table 3) indicate that students’ attitudes toward most categories are positive

Four out of six categories that relate to the process of science have a higher number of positive comments than negative ones (Fig. 5). They include “independence,” “experimental design,” “connection to a broader picture,” and “iterative process.” The “independence” category is the most commented on among the items related to the process of science (12.96% of all responses, 62% positive). Students also generally reported positive reactions to “experimental design” (60.4%). “Connection to a broader picture,” and “iterative process” are the least commented upon items (3.6% and 3.1%, respectively), which is not surprising since these aspects of research are likely to be less obvious to novice scientists (Fig. 5).

“Working with uncertainty” had the largest proportion of negative comments (68%) overall (Fig. 5). 24% of students perceived this category positively, citing that they enjoyed the process of discovering something new or running experiments that had not been published. However, this part of scientific work made a number of students uncomfortable or frustrated, consistent with literature that suggests students experience frustration with ambiguity (Yerushalmi et al. 2007). This idea also explains some of the students’ negative comments that fell under “independence” (27% negative). These comments were mixed; some students wished for more independence while others wanted more guidance and structure. Often we saw these comments within the same module (from a module on shark tooth biomechanics: “I dislike the restrictions we had on our topics due to limited teeth selection,” “a little too open ended”).

One of the other three areas with more negative (59%) than positive comments was “collaboration.” These comments often focused on group members who were perceived to do less work than the other members of the group. The issue of unequal distribution of workload in group projects is not specific to FSBio 201. Since science is a highly collaborative process, we learn from these data that we should mentor students in how to productively manage group dynamics. One such way that has been introduced in some modules is to implement student assessment of both their own contributions to the independent experiment and the contribution of the other group members. However, additional mentoring at the start of the semester would be helpful.

#### RSE data on communication skills show that students’ perception align with learning outcomes on science communication (LO 1, 3)

Students commented most frequently on the writing component of the course (24.2% of total responses) (Fig. 5). 63% of those responses were positive. Students frequently mentioned their appreciation of the highly structured, scaffolded writing process, the general feedback given to them, and their impression that they perceived themselves as becoming better writers over the course of the semester. These self-reported comments are in line with students’ perceptions reported in the CURE survey. Given that improved writing within the discipline is one of the student learning outcomes for both FSBio 201 and the overall Allegheny FS curriculum, we find this result especially encouraging. Student comments on oral communication were less frequent (13.2% of total responses) than those for writing but still mostly positive (76%). Students frequently mentioned appreciation of practice talks and feedback from instructors. Negative comments related to written and oral communication most commonly asked for more explicit guidance.

#### RSE data on peer-to-peer teaching suggest that formal training will be beneficial for teaching assistants

Peer mentoring comments (51% negative) were mostly about the quality of feedback on writing drafts given by the undergraduate teaching assistants (Fig. 5). Students who made negative comments cited a lack of congruence between teaching assistant feedback on drafts and instructor feedback on the final paper, whereas positive feedback noted that teaching assistant feedback resulted in high grades on the final paper. When we choose teaching assistants for FSBio 201, we look for students with strong writing skills as evidenced by work completed for FSBio 201 and other courses. Therefore, we do not believe a lack of skill on the part of the teaching assistants has led to the negative comments. Instead, we, as instructors, likely need to spend more time mentoring our teaching assistants and finding ways to more clearly communicate our own final paper expectations to the teaching assistants, as they are providing feedback on initial paper drafts. While we do employ a standard checklist to aid in TA-provided feedback, we might address this set of student concerns by more formal training for the teaching assistants and by clarifying students’ expectations for the purpose, goals, and limitations of this form of peer mentoring.

### Summary

Together, students’ self-reported responses in the CURE survey and RSE results indicate that the FSBio 201 learning outcome that aligns with students’ perceptions most closely is the one focused on science communication skills. Students perceived that they gained both writing and oral communication skills through FSBio 201. Students believed that they were learning “how to do” science and that there is more to it than performing an experiment and shouting “Eureka!.” They also exhibited positive reactions toward different aspects of research such as how to plan and execute an experiment, and how to interpret their results in the context of the scientific literature. These are all skills that will be important as they move toward their senior project research. Just as important, they appeared to be learning lessons that are not part of a textbook: that science is messy, can be tedious, involves regular collaboration, and may not always have a right answer. The students may not like these particular lessons, but their self-reported responses indicate that they appear to be learning them nonetheless:

“Although hard to first get a grasp on, your module allowed me to face the reality that most researchers are partially in the dark for some of their experiments, but it’s ok not to know everything as the results of an experiment can help one really understand how a process works, even with a missing piece or two.”

## Pandemic pedagogy: shifting to virtual and hybrid classrooms

Spring 2020 FSBio 201 began with the three-module approach, but when the global Covid-19 pandemic forced us to suspend in-person teaching 9 weeks into the 14-week semester, the instructors pivoted from the typical laboratory experiences to a remote final module. The remote module emphasized reading and working with literature, data analysis, and experimental design. Students read an early paper linking cigarette smoking with lung cancer (Wynder and Graham 1950) and answered guiding questions. Students then graphed recent data from the CDC on smoking rates for men and women and diagnoses of lung cancer broken down across demographics, compared the older and recent datasets, and proposed ideas for future studies on the general topic of smoking and lung cancer. This module was a pared-down version of what our epidemiologist colleague had previously taught in the course. Allegheny did not officially administer student evaluations in Spring 2020, but instructors collected evaluations on a voluntary basis. Although the students reported appreciating regular communication from the instructors and the ability to continue to learn novel material, several students indicated that they would have appreciated even more engagement in the remote module. In future iterations of remote delivery, students could find CDC data to address a research question or hypothesis of their own, which would enable similar types of written and oral assignments as other modules.

The Fall 2020 semester had its own unique challenges. Allegheny held in-person classes, but students were able to choose whether to attend classes in-person or virtually. Hence, all classes needed to accommodate students both in the classroom and participating via video conferencing. The two instructors specifically designed their modules to enable both formats, with minimal hands-on laboratory time. One module examined the relationship between single nucleotide polymorphism variation and phenotype in dogs (Hultman and Mellgren 2014), while the other examined the gut microbiome of the flour beetle *Callosobruchus maculatus*. In both modules, lab groups consisted of a mix of in-person and remote students for an integrated experience. In-person students performed one or two short experiments, typically with remote students observing via video. All members of the groups worked together to analyze data, prepare presentations, and develop follow-up experiments. Since the two-module format already emphasized communication skills and interpreting the scientific literature, only a moderate shift was necessary to address the hybrid guidelines for the semester.

Despite the disruptions to FSBio 201 during the two 2020 semesters—requiring abrupt, unexpected reorganization in the spring, and complicated hybrid considerations in the fall—the existing structure, goals, and philosophy of the course allowed for successful delivery of a CURE-focused student experience. In addition, the increased flexibility and creativity demonstrated by the new modules developed in both semesters adds to the versatility of the course, exposing students to entirely new formats of experimental biology.

## What to know before you run the course

Based on our experience with FSBio 201, we provide a basic framework for developing a CURE course. In addition to the general framework, we provide a few practical suggestions. First, we recommend that the course be modified to fit your institution’s particular curricular needs. Our learning outcomes and formative and summative assessments work well for Allegheny’s curriculum, which scaffolds communication and research throughout all 4 years and includes developing students’ oral and written communication skills prior to FSBio 201. The detailed organization of the course can be found under “Course organization and mechanics.”

Our next set of recommendations concern the experiments themselves. After defining learning outcomes based on your curricular needs, we recommend including at least two iterations of the planned experiment. The first experiment, planned by the instructor, provides initial exposure to the technique or process of data collection. The second one (and following experiments, if applicable) provides opportunities for students to design their own experiments. It is crucial to find an experimental preparation that is simple, yet intriguing and relatable enough for students to engage in the process of research and focus on communication. At the same time, make sure there are plenty of creative directions to go with the project to give students the freedom to design their independent projects, while being mindful of potential challenges in navigating literature and technology.

A significant amount of meta-teaching needs to occur in this type of course to help the students through what is likely their first experience with scientific research, which can be overwhelming and/or exhilarating for them. A concept that cannot be emphasized enough is that an unsupported hypothesis is not the same thing as a wrong answer. Students who associate success in science with obtaining the “right” answer may view data at odds with their expectations as experimental failure, rather than appreciating that unexpected outcomes, and the consequent need to refine one’s model, are a crucial part of scientific progress. Intentionally preparing students for these challenges is key to sustaining their enthusiasm and engagement.

Students should become aware through the course that different fields within biology have different approaches when it comes to writing. This awareness can develop from reading and analyzing different styles of papers (an ecology paper and a molecular biology paper look *very* different from one another) and from writing their own papers on different topics. Reassure the students that the differences they observe among instructors and modules are real and relevant.

Finally, this type of course is high-engagement for both the student and the instructor. Students commented frequently in the RSEs on how important instructor enthusiasm and energy were to them. Instructors need to guide students in all phases of their experiments without restricting their independence. Regular constructive feedback is incredibly important as students move through designing experiments, analyzing data, and writing and speaking. We emphasize the importance of formative assessments for the success of the CURE course, as indicated by students’ appreciation for opportunities to practice their communication skills and frequent feedback on these formative assessments. It helps, particularly when in the midst of time-consuming assessment tasks, to take the long view of the course’s value. Effort invested in laying a strong foundation at this stage of students’ scientific development benefits them in later years, when they can combine these essential skills with growing expertise in the field to take on more complex tasks.

## Supplementary Material

icab128_Supplemental_Files

## Data Availability

The raw data underlying this article cannot be shared publicly to maintain the privacy of the individuals who participated in this study. Deidentified data will be shared on reasonable request to the corresponding author.

## Notes

From the symposium “Biology Beyond the Classroom: Experiental Learning Through Authentic Research, Design, and Community Engagement” presented at the annual meeting of the Society for Integrative and Comparative Biology virtual annual meeting, January 3–February 28, 2021.
