# Supplementary material for: Students’ Perceptions of FSBio 201, A CURE-Based Course that Scaffolds Research and Scientific Communication, Align with Learning Outcomes
Source: Integr Comp Biol. 2021 Jun 10;61(3):944–56. doi: 10.1093/icb/icab128 (PMC8490692; doi:10.1093/icb/icab128)
Supplement: icab128_Supplemental_Files [file icab128_supplemental_files.zip › icb-2021-0064-File008.docx]

**Supplement 1: Description of FSBio 201 modules**

| **Module Topic** | **Brief Description** | **Common Variations** | **Associated Resources** |
| --- | --- | --- | --- |
| Plant peroxidase isoenzyme analysis | Peroxidases are extracted from plants treated in various ways and analyzed by native gel electrophoresis. Activity levels and isoenzyme patterns are assessed by staining with a peroxidase substrate. | Plant species, extracted tissues, developmental stage, growth conditions; treatments with various stressors/chemicals. | Based on "IND-22 (Characterization of Peroxidases in Plants)" from Modern Biology Inc. |
| UV mutagenesis & repair in *Serratia marcescens* | A UV crosslinker is used to damage *Serratia marcescens*; mutation frequency is monitored using the pigment prodigiosin | Attempt to protect cells from damage with sunscreen or another chemical; compare light repair to dark repair. | Adapted from a lab exercise developed by Bruce A. Voyles, Professor Emeritus at Grinnell College  Labrum EL, Bunting MI.1953. Spontaneous and induced color-variation of the HY strain of *Serratia marcescens*. Journal of Bacteriology. 65(4):394-404.  Morrison DA. 1966. Prodigiosin synthesis in mutants of *Serratia marcescens*. Journal of Bacteriology. 91(4):1599-1604. |
| *Dictyostelium discoideum* chemotaxis | Chemotaxis of starved *Dictyostelium discoidem* cells, moving across non-nutrient agar towards a well of cAMP, is monitored via a stereo zoom dissecting microscope. | Altering the starvation time; changing the concentration of cells or cAMP; using inhibitors to affect cAMP secretion or stability; assessing the effect of mutations in components of the associated signaling pathways. | Chemotaxis assay based on that described in:  Wallace LJ, Frazier, WA. 1979. Photoaffinity labeling of cyclic-AMP- and AMP-binding proteins of differentiating *Dictyostelium disciodeum* cells. Proceedings of the Natural Academy of Sciences. 76(9):4250-4254 .  Nandini-Kishore SG, Frazier WA. 1981. [3H]Methotrexate as a ligand for the folate receptor of *Dictyostelium discoideum*. Proceedings of the Natural Academy of Sciences. 78(12):7299-7303. |
| Dog SNP genotyping | PCR analysis and restriction digests are used to identify SNP variations potentially associated with physical/behavioral traits in dogs. | Choice of traits and breeds to investigate; can also be expanded to phylogenetics or analysis of forensic scenarios. | Based on: Hultman K, Mellgren E. 2014. Fetching SNPs: A Dog Genotyping Laboratory for Undergraduate Biology. Genetics Society of America Peer-Reviewed Education Portal (GSA PREP): 2014.001; doi: 10.1534/gsaprep.2014.001 |
| Using DNA barcodes to assess insect & plant diversity | After collection of plant or insect samples from campus, an initial attempt is made to identify the organisms based on morphology (via a dichotomous key/online resources), followed by use of DNA barcoding as a second means of identification. | No variable/variation beyond initial choice of organism; instead module is used to introduce exploratory/discovery (vs. hypothesis-driven) research. The investigation focuses more on the challenges of using morphology alone for species identification. | Part of a DNA Learning Center collaborative project, as described via DNA Learning Center: DNA Barcoding 101. Cold Spring Harbor NY: Cold Spring Harbor Laboratory Learning Center; [accessed 2021 Jan 15]. <https://dnabarcoding101.org/about/> |
| Genes and environment interaction (*Saccharomyces cerevisiae*) | The effect of a mutation on the response to different environments (*e.g.,* UV radiation, caffeine, Indole-3-Carbonol) is determined by measuring the growth of wild-type and mutant *S. cerevisiae* strains via serial dilution and colony counts. The genes studied in this lab are functionally conserved in humans. | Different temperature-sensitive mutants; various treatments. |  |
| Cancer cell biology | The effect of treatment with a selected chemical (chosen from a list of options) on the survival of mammalian cancer cells is quantitated via a hemacytometer. | Drug and exposure time. |  |
| Teratogens & zebrafish development | Zebrafish embryos are treated with retinoic acid and the effects on organismal development observed. | Vary time and concentration; use other compounds (*e.g.*, ethanol, pesticide). |  |
| Bean beetle (*Callosobruchus maculatus*) microbiomes | Bean beetles are crushed and their gut contents grown on selective agar; in initial semester student projects focused on a bean beetle microbiome database, but could be expanded into more work with the beetles themselves in the future. | Factors that may affect bacterial numbers or types of bacteria present in the gut (sex, food source, selective agar used). | Designed using materials related to the Bean Beetle Microbiome Project, available via Bean Beetles: A Model Organism for Inquiry-Based Undergraduate Laboratories. c2005-2020. Blumer LS, Beck CW; [accessed 2021 Jan 14]. <https://www.beanbeetle.org/microbiome/resources-for-online-teaching/> |
| Crayfish muscle physiology | After simple dissection, crayfish heart rate is monitored. Students apply a variety of pharmacological manipulations and compare heart rates to the control (no manipulation). | Typical chemicals used include epinephrine, caffeine, dopamine, AChR blockers. |  |
| Human dive response | ECG measurements are taken on fellow students before, during, and after submersion of the face in cold water. A drop in heart rate indicates a dive response. | Testing parameters of the response such as water temperature, physical fitness, caffeine, necessity of wetness and/or face submersion. |  |
| Shark tooth biomechanics | The force necessary to puncture various dead prey items with various shark teeth is measured using kitchen scales. | Shark teeth from different species, shark teeth from one species but different locations in the jaw, different prey items. | Based on Bergman, JN, Lajuenesse ML, Motta PJ. 2017. Teeth penetration force of the tiger shark *Galeocerdo cuvier* and sandbar shark *Carcharhinus plumbeus*. Journal of Fish Biology. 91: 460-472. |
| Salamander jumping kinematics | High-speed videography is used to film plethodontid salamanders jumping. Students measure height, velocity, angular velocity of bending. | Different plethodontid salamander species, different temperatures, different heights, different environments (e.g. leaf matter, color, predators). | Based on Ryerson WG, Hessel AL, Whitenack LB. 2016. Comparative jumping mechanics in plethodontid salamanders. Journal of Zoology 299: 51-57. |
| Mating behavior and maternal care in the ring-legged earwig (*Euborellia annulipes*) | Initial assessment of (1) costs and benefits of maternal care, and (2) role of body size in male-male competition. | Manipulate number of male competitors, earwig size, presence of egg-predators, temperature, and other environmental parameters. |  |
| Physiological ecology of plethodontid salamanders | Oxygen-sensing equipment is used to measure salamander metabolic rate; feeding behavior serves as a second proxy. | Manipulate environmental variables (photoperiod, temperature, food availability, etc.) |  |
| Goldenrod gall ecology | Fieldwork-based analysis of goldenrod gall system in which *Solidago* goldenrod stems are parasitized by *Eurosta solidaginis* gallflies, gallfly larvae are parasitzed by *Eurytoma gigantea* and *Eurytoma obtusiventris* wasps are preyed on by *Mordellistena* beetles, and avian predators prey on insect gall contents. Additional gall formers (*Rhopalomyia solidaginis* bunch gall flies and, occasionally, *Gnorimoschema gallaesolidaginis* spindle gall moths) can also be studied | Presence/absence of the various gall formers, insect parasites, and bird predation; gall size and location on stem, etc. can be analyzed in response to each other or to environmental conditions. | Based on materials at *Solidago Eurosta* Gall: A Resource for Teaching and Research.c2021. Lewisburg, PA: Abrahamson W; [accessed 2021 Jan 15]. <http://solidago.scholar.bucknell.edu/> |
| Phytoplankton community structure | Changes in the community structure of an *in vitro* simulated phytoplankton community, containing six species of algae from commercial suppliers, are analyzed (identification & quantitation via light microscopy) | Alter conditions such as light (presence/absence, intensity, and wavelengths), pH, concentration of various nutrients (*e.g.*, nitrogen, phosphorous), herbivory by zooplankton such as *Daphnia* waterfleas. |  |
| Epidemiology | Existing epidemiological data are used to explore association between diseases and other factors. | Students are given latitude in choice of disease and factor(s). |  |
