# Supplementary material for: Students’ Perceptions of FSBio 201, A CURE-Based Course that Scaffolds Research and Scientific Communication, Align with Learning Outcomes
Source: Integr Comp Biol. 2021 Jun 10;61(3):944–56. doi: 10.1093/icb/icab128 (PMC8490692; doi:10.1093/icb/icab128)
Supplement: icab128_Supplemental_Files [file icab128_supplemental_files.zip › icb-2021-0064-File009.docx]

**Supplement 2:** Post-course mean scores for all categories measured in the CURE survey. From Spring 2014 - Spring 2017, 210 Allegheny students and 41,659 students in total (“All Students”, including the 210 Allegheny students) completed the post-course survey. Unpaired t-tests were performed to determine statistical significance (Allegheny n = 9 semesters, All Students n = 5 years, df = 12, ɑ = 0.05, * = statistically significant with significance levels adjusted with Holm’s Sequential Bonferroni method within each of the 3 categories). Categories in each table are sorted by difference between Allegheny students and All Students, such that items on which Allegheny students scored lower than All Students with the largest difference between means are at the top of the table. The “bottom five”, representing the largest difference with low Allegheny student scores, are shaded in grey. The “top five”, representing the largest difference with high Allegheny student scores, are shaded in yellow.

| ***Course Elements*** | **Allegheny mean score** | **All Students mean score** | **Difference between means** | **Allegheny STDEV** | **All Students STDEV** | **t-value** | **p-value** |
| --- | --- | --- | --- | --- | --- | --- | --- |
| **Take tests in class** | 2.118 | 3.314 | -1.196 | 0.594 | 0.046 | 4.412 | 0.0008* |
| **Maintain lab notebook** | 2.644 | 3.540 | -0.896 | 0.551 | 0.066 | 3.556 | 0.0040 |
| **Read a textbook** | 2.270 | 3.070 | -0.800 | 0.513 | 0.060 | 3.411 | 0.0052 |
| **Work on problem sets** | 2.691 | 3.422 | -0.731 | 0.312 | 0.035 | 5.136 | 0.0002* |
| **Present posters** | 2.891 | 3.318 | -0.427 | 0.548 | 0.082 | 1.702 | 0.1145 |
| **Listen to lectures** | 3.202 | 3.518 | -0.316 | 0.190 | 0.098 | 3.428 | 0.0050 |
| **Work as a whole class** | 2.970 | 3.240 | -0.270 | 0.262 | 0.052 | 2.244 | 0.0444 |
| **Lab or project where only instructor knows outcome** | 3.220 | 3.412 | -0.192 | 0.321 | 0.026 | 1.311 | 0.2144 |
| **Computer modeling** | 2.998 | 3.118 | -0.120 | 0.239 | 0.045 | 1.093 | 0.2957 |
| **Scripted lab or project where students know outcome** | 3.178 | 3.286 | -0.108 | 0.231 | 0.053 | 1.013 | 0.3309 |
| **Work individually** | 3.328 | 3.404 | -0.076 | 0.339 | 0.042 | 0.491 | 0.6321 |
| **Critique work of other students** | 3.268 | 3.294 | -0.026 | 0.358 | 0.084 | 0.159 | 0.8765 |
| **Discuss reading materials in class** | 3.543 | 3.548 | -0.005 | 0.309 | 0.062 | 0.103 | 0.9195 |
| **A least one project assigned and structured by instructor** | 3.817 | 3.652 | 0.165 | 0.253 | 0.073 | 1.401 | 0.1865 |
| **Work in small groups** | 4.162 | 3.898 | 0.264 | 0.192 | 0.074 | 2.918 | 0.0129 |
| **Analyze data** | 4.316 | 4.040 | 0.276 | 0.161 | 0.062 | 3.616 | 0.0035 |
| **Become responsible for a part of the project** | 4.208 | 3.932 | 0.276 | 0.163 | 0.083 | 3.505 | 0.0043 |
| **Collect data** | 4.239 | 3.930 | 0.309 | 0.163 | 0.096 | 3.838 | 0.0024* |
| **A project where students have input into process or topic** | 4.352 | 3.860 | 0.492 | 0.231 | 0.070 | 4.576 | 0.0006* |
| **Lab or project where no one knows the outcome** | 3.989 | 3.394 | 0.595 | 0.352 | 0.128 | 3.592 | 0.0037 |
| **Write a research proposal** | 4.082 | 3.484 | 0.598 | 0.141 | 0.102 | 8.302 | <0.0001* |
| **Present results in written papers or reports** | 4.421 | 3.748 | 0.673 | 0.167 | 0.095 | 8.202 | <0.0001* |
| **Present results orally** | 4.319 | 3.626 | 0.693 | 0.221 | 0.082 | 6.661 | <0.0001* |
| **Read primary scientific literature** | 4.344 | 3.592 | 0.752 | 0.161 | 0.122 | 9.059 | <0.0001* |
| **A project entirely of student design** | 4.337 | 3.556 | 0.781 | 0.212 | 0.065 | 7.910 | <0.0001* |

| ***Learning Gains*** | **Allegheny mean score** | **All Students mean score** | **Difference between means** | **Allegheny STDEV** | **All Students STDEV** | **t-value** | **p-value** |
| --- | --- | --- | --- | --- | --- | --- | --- |
| **Clarification of a career path** | 2.622 | 3.050 | -0.428 | 0.225 | 0.139 | 3.821 | 0.0024* |
| **Learning ethical conduct** | 3.100 | 3.264 | -0.164 | 0.278 | 0.159 | 1.202 | 0.2525 |
| **Learning to work independently** | 3.299 | 3.410 | -0.111 | 0.252 | 0.096 | 0.935 | 0.3681 |
| **Becoming part of a learning community** | 3.644 | 3.526 | 0.118 | 0.292 | 0.134 | 0.848 | 0.4132 |
| **Learning laboratory techniques** | 3.906 | 3.780 | 0.126 | 0.233 | 0.072 | 1.158 | 0.2695 |
| **Confidence in my potential as a teacher** | 3.152 | 3.022 | 0.130 | 0.264 | 0.124 | 1.029 | 0.3237 |
| **Understanding how knowledge is constructed** | 3.636 | 3.504 | 0.132 | 0.238 | 0.116 | 1.146 | 0.2741 |
| **Ability to integrate theory and practice** | 3.660 | 3.522 | 0.138 | 0.137 | 0.088 | 2.021 | 0.0662 |
| **Understanding science** | 3.830 | 3.662 | 0.168 | 0.200 | 0.104 | 1.732 | 0.1089 |
| **Understanding how scientists think** | 3.654 | 3.480 | 0.174 | 0.189 | 0.123 | 1.840 | 0.0906 |
| **Tolerance for obstacles faced in the research process** | 3.790 | 3.566 | 0.224 | 0.174 | 0.116 | 2.553 | 0.0253 |
| **Understanding how scientists work on real problems** | 3.881 | 3.646 | 0.235 | 0.280 | 0.117 | 1.766 | 0.1027 |
| **Self-confidence** | 3.587 | 3.314 | 0.273 | 0.364 | 0.151 | 1.579 | 0.1402 |
| **Understanding the research process** | 3.831 | 3.552 | 0.279 | 0.142 | 0.133 | 3.593 | 0.0037 |
| **Understanding that scientific assertions require supporting evidence** | 3.978 | 3.692 | 0.286 | 0.239 | 0.092 | 2.529 | 0.0265 |
| **Skill in interpretation of results** | 3.887 | 3.600 | 0.287 | 0.184 | 0.107 | 3.157 | 0.0083 |
| **Ability to analyze data and other information** | 4.078 | 3.774 | 0.304 | 0.310 | 0.088 | 2.110 | 0.0566 |
| **Readiness for more demanding research** | 3.846 | 3.488 | 0.358 | 0.224 | 0.119 | 3.277 | 0.0066 |
| **Ability to read and understand primary literature** | 4.132 | 3.440 | 0.692 | 0.210 | 0.157 | 6.398 | <0.0001* |
| **Skill in how to give an effective oral presentation** | 4.122 | 3.248 | 0.874 | 0.363 | 0.136 | 5.108 | 0.0003* |
| **Skill in science writing** | 4.322 | 3.430 | 0.892 | 0.216 | 0.141 | 8.235 | <0.0001* |

| ***Attitudes About Science*** | **Allegheny mean score** | **All Students mean score** | **Difference between means** | **Allegheny STDEV** | **All Students STDEV** | **t-value** | **p-value** |
| --- | --- | --- | --- | --- | --- | --- | --- |
| **I wish science instructors would just tell us what we need to know so we can learn it** | 2.444 | 2.924 | -0.480 | 0.292 | 0.034 | 3.600 | 0.0036 |
| **There is too much emphasis in science classes on figuring things out for yourself** | 2.330 | 2.792 | -0.462 | 0.267 | 0.022 | 3.789 | 0.0026* |
| **If an experiment shows that something doesn't work, the experiment was a failure** | 1.519 | 1.970 | -0.451 | 0.233 | 0.037 | 4.169 | 0.0013* |
| **Scientists know what the results of their experiments will be before they start** | 1.724 | 2.168 | -0.444 | 0.185 | 0.042 | 5.211 | 0.0002* |
| **Science is not connected to non-science fields such as history, literature, economics, or art** | 1.846 | 2.200 | -0.354 | 0.159 | 0.019 | 4.864 | 0.0004* |
| **Only scientific experts are qualified to make judgments on scientific issues** | 2.217 | 2.568 | -0.351 | 0.202 | 0.048 | 3.758 | 0.0027* |
| **Creativity does not play a role in science** | 1.757 | 2.080 | -0.323 | 0.148 | 0.016 | 4.787 | 0.0004* |
| **Students who do not major/concentrate in science should not have to take science courses** | 2.239 | 2.498 | -0.259 | 0.123 | 0.019 | 4.589 | 0.0006* |
| **Science is essentially an accumulation of facts, rules, and formulas** | 2.967 | 3.184 | -0.217 | 0.311 | 0.086 | 1.506 | 0.1578 |
| **Scientists play with statistics to support their own ideas** | 2.646 | 2.854 | -0.208 | 0.267 | 0.032 | 1.711 | 0.1129 |
| **When scientific results conflict with my personal experience, I follow my experience in making choices** | 2.961 | 3.132 | -0.171 | 0.191 | 0.030 | 1.956 | 0.0742 |
| **Real scientists don't follow the scientific method in a straight line** | 3.126 | 3.254 | -0.128 | 0.220 | 0.015 | 1.282 | 0.2241 |
| **When experts disagree on a science question, it's because they don't know all the facts yet** | 2.940 | 3.048 | -0.108 | 0.277 | 0.024 | 0.854 | 0.4096 |
| **You can rely on scientific results to be true and correct** | 3.267 | 3.360 | -0.093 | 0.260 | 0.084 | 0.770 | 0.4565 |
| **Lab experiments are used to confirm information studied in science class** | 3.746 | 3.676 | 0.070 | 0.193 | 0.047 | 0.779 | 0.4510 |
| **Since nothing in science is known for certain, all theories are equally valid** | 2.867 | 2.760 | 0.107 | 0.367 | 0.073 | 0.632 | 0.5394 |
| **Main job of the instructor is to structure the work so that we can learn it ourselves** | 3.497 | 3.378 | 0.119 | 0.327 | 0.031 | 0.794 | 0.4427 |
| **I can do well in science courses** | 4.196 | 4.012 | 0.184 | 0.144 | 0.044 | 2.732 | 0.0182 |
| **The process of writing in science is helpful for understanding scientific ideas** | 4.171 | 3.960 | 0.211 | 0.160 | 0.090 | 2.697 | 0.0194 |
| **Explaining science ideas to others has helped me understand the ideas better** | 4.336 | 4.084 | 0.252 | 0.198 | 0.045 | 2.759 | 0.0173 |
| **I get personal satisfaction when I solve a scientific problem by figuring it out myself** | 4.438 | 4.184 | 0.254 | 0.242 | 0.036 | 2.292 | 0.0408 |
| **Even if I forget the facts, I'll still be able to use thinking skills learned in science** | 4.444 | 4.160 | 0.284 | 0.104 | 0.064 | 5.487 | 0.0010* |
