# Supplementary material for: Students’ Perceptions of FSBio 201, A CURE-Based Course that Scaffolds Research and Scientific Communication, Align with Learning Outcomes
Source: Integr Comp Biol. 2021 Jun 10;61(3):944–56. doi: 10.1093/icb/icab128 (PMC8490692; doi:10.1093/icb/icab128)
Supplement: icab128_Supplemental_Files [file icab128_supplemental_files.zip › icb-2021-0064-File010.docx]

*End-of-semester evaluations (RSEs): extensive description of RSEs and coding framework, expanded results.*

Description of RSE forms

Like most colleges and universities, Allegheny College employs surveys (Report of Student Experience, or RSEs) at the end of each semester for students to provide feedback to their instructors. We analyzed RSEs from the Fall 2013 through Fall 2019 semesters, using data from two different versions of the RSE form. The standard College form uses two prompts: “Please identify the aspects of this course that were most useful in helping you learn, and briefly explain why you found them helpful” and “Please identify the aspects of this course that were least helpful in helping you learn, and briefly explain why they were not helpful and how they could be made to be more helpful.” The prompt on the FSBio 201-specific form was “For each of the modules in which you participated, please comment briefly on what you liked and disliked about the module in question. If you were in a position to change this project, what changes would you make and why?”, and included sections for “General” (the course as a whole) and for each instructor.

Development and implementation of the coding framework

To develop a framework for coding the Reports of Student Experience (RSEs), we first evaluated RSEs from five semesters and established several categories. We refined the definition of each category after deliberation among the co-authors to finalize the coding guidelines. To assess the reliability of the guidelines, 18 comments randomly chosen from different semesters were coded by all co-authors (see description below). Approximately 70-80% of the comments were coded the same way by the majority of the coders in each category. Majority was defined as not having more than one outlier in each category. Coders met to discuss the results of this preliminary analysis and to further clarify and establish the finalized coding guidelines. One author (LBW) was also tasked with examining the final data set for inconsistencies among coders and resolving any RSE data that were tagged by coders as needing adjudication. This preliminary analysis coupled with our subsequent discussion demonstrated that the coding guidelines carry sufficient clarity to yield reasonably consistent data regardless of the person who is coding.

The variables we coded fall into three broad categories: 1) content specific to a module (organisms/experimental subjects, biological processes/sub-disciplines, and experimental approach/techniques specific to a sub-discipline); 2) different facets of research universal to all sub-disciplines (designing experiments, iterative process, independence, working with uncertainty or recognizing the aspect of discovery, collaboration, and making connections to a broader picture); 3) communication skills (oral presentation and writing a research paper) (Table 3). Measuring these variables allows us to assess how closely students’ perceptions and the learning outcomes of FSBio 201 align. In addition, we included a category to indicate students’ attitudes towards peer-to-peer learning from teaching assistants and two categories that assessed students’ overall response to the module or course (desire to further student’s interest and general likes/dislikes). We used the same coding scheme for each category (positive, negative, neutral). Both general comments and module-specific comments were coded using the same guidelines. An individual comment could be scored across multiple categories. For example, if a student stated “I liked working with zebrafish, but not with the microscopes” we counted this comment towards “model organism” (positive) and “experimental approach” (negative).

To minimize subjectivity, the authors did not code RSEs from their own modules. Only one coder was responsible for a given semester’s set of RSEs. Due to this limitation, we did not perform any statistical analyses and present the RSE results as qualitative data. We coded RSEs from 19 consecutive semesters (Fall 2013-Fall 2019) that encompassed 48 total modules and 1551 individual comments. Because the FSBio 201-specific form had up to four sections (General plus three instructors) and college RSEs are completed for individual instructors (up to three separate instructors per student), we analyzed more comments than students enrolled. The categories and codes are described in detail, with representative examples, in Supplement 4. Protocols for this part of the study were approved by the Allegheny College Institutional Review Board (protocol 2020-05).

Expanded results:

*RSE data generally show positive perceptions of the course in a majority of the surveyed categories*

Because the RSE prompts are open-ended and are not explicitly limited to our coding categories, we identified a large number of comments that did not fit our coding scheme (37.0% of total responses) (Table 4 in manuscript). These comments included personal attributes of the instructor and comments on speed of the return of work. One theme that emerged from the “other” category was the importance of instructor enthusiasm and energy. Instructor enthusiasm can motivate students (Alsharif 2011), and the RSE data suggest conveying passion for science may positively change the perception of FSBio 201. In general, student comments on the RSEs demonstrate a positive attitude towards FSBio 201. Ten out of thirteen categories had more positive comments than negative ones, ranging from 54% (iterative process) to 84% positive (general like/dislike of the course or module) (Fig. 5 in manuscript).

*Through students’ self-reported RSE data on content specific to a module, we infer that students gain a general understanding of biological research (LO1)*

To gain a better understanding of student attitudes about particular modules and subfields, we more closely examined three categories: “model organism,” “biological process,” and “experimental approach.” As a department, we anecdotally perceive certain themes from our students, such as “I hate plants,” “I’m going to medical school, so learning about sharks won’t benefit me,” “I just don’t get math and computers,” and others. Examining these three categories provides the opportunity to assess the validity of those anecdotal perceptions. Because some modules are taught more frequently than others, we did not attempt to compare how these categories are distributed across modules. Instead, we have looked at the broad applicability of these categories for the course as a whole.

When we examined mentions of specific model organisms, no broad taxonomic group had more negative than positive comments (Supplement 5). This result held true whether mentions of humans were categorized separately or with other animals. Only 8.2% of comments fell under “biological process”, and comments were largely positive (66%). We could not find a specific trend under “biological process” as students used a wide range of vocabularies to describe the same biological process. Self-reported data on another module-specific category, “experimental approach”, reveal sub-discipline specific practices that students enjoy or do not enjoy. These data also suggest that students are gaining a more realistic view of how science is done. For example, total responses about fieldwork were 26.4% of all “experimental approach” responses, and 92.9% of those were positive. Biology programs, including our own, sometimes perceive that a high percentage of biology majors are pre-health students and that they are interested only in disease-related research. Students’ reported appreciation for fieldwork suggests that these perceptions are not accurate. On the other hand, two aspects within “experimental approach” that generated negative perceptions were (i) coming in outside of class time (92.9% negative) and (ii) having to wait for procedures to run (90.9% negative). Working scientists understand the inevitability of these kinds of inconveniences (when has a biologist *ever* made good on their promise to only be stopping into the lab for five minutes?), and it is not surprising that students might have a negative reaction to their initial exposure to this scientific reality. Overall, data in these categories provide us the metrics to gauge students’ perception of the process of science (LO1) not only as a way of approaching a problem but also as an undertaking in which specific common practices are followed to achieve a successful outcome (Table 1 in manuscript). These realistic experiences are, we hope, informative for students who are considering a career in science.

*RSE data on scientific process (different facets of research universal to all sub-disciplines in Table 3) indicate that students’ attitudes towards most categories are positive*

Four out of six categories that relate to the process of science have a higher number of positive comments than negative ones (Fig. 5 in manuscript). They include “independence,” “experimental design,” “connection to a broader picture,” and “iterative process.” The “independence” category is the most commented on among the items related to the process of science (12.96% of all responses, 62% positive). Students also generally reported positive reactions to “experimental design” (60.4%). As we looked at more specific themes within this category, we found modules with heavy reliance on computers and statistics had the most frequent and most negative comments (40.5% positive, 53.4% negative) (Supplement 5). Positive comments mentioned that they appreciated tutorials or looked toward future work beyond FSBio 201. Negative comments tended to focus on the associated complexity, either of the equipment, the tasks, or the interpretations. “Connection to a broader picture,” and “iterative process” are the least commented upon items (3.6% and 3.1%, respectively), which is not surprising since these aspects of research are likely to be less obvious to novice scientists (Fig. 5).

“Working with uncertainty” had the largest proportion of negative comments (68%) overall (Fig. 5 in manuscript). 24% of students perceived this category positively, citing that they enjoyed the process of discovering something new or running experiments that had not been published. However, this part of scientific work made a number of students uncomfortable or frustrated, consistent with literature that suggests students experience frustration with ambiguity (Yerushalmi et al. 2007). Students who commented negatively on this topic often talked about how they could not find the “right answer” in the literature. If we consider this result in the larger STEM education landscape, this finding is not surprising. Science is often taught as a series of facts, processes to memorize, and cookbook-type labs (NRC 2003, Yerushalmi et al. 2007). Even accounts of famous experiments do not show the failures or messiness involved. We talk about scientists “discovering DNA” , for example, but fail to highlight that if we are discovering it, it was previously unknown. Moving from this familiar world of knowns and facts can be jarring and uncomfortable. This idea also explains some of the students' negative comments that fell under “independence” (27% negative). These comments were mixed; some students wished for more independence while others wanted more guidance and structure. Often we saw these comments within the same module (from a module on shark tooth biomechanics: “I dislike the restrictions we had on our topics due to limited teeth selection”, “a little too open ended”).

One of the other three areas with more negative (59%) than positive comments was “collaboration.” These comments often focused on group members who were perceived to do less work than the other members of the group. The issue of unequal distribution of workload in group projects is not specific to FSBio 201. Since science is a highly collaborative process, we learn from these data that we should mentor students in how to productively manage group dynamics. One such way that has been introduced in some modules is to implement student assessment of both their own contributions to the independent experiment and the contribution of the other group members. However, additional mentoring at the start of the semester would be helpful.

*RSE data on communication skills show that students’ perception align with learning outcomes on science communication (LO 1, 3)*

Students commented most frequently on the writing component of the course (24.2% of total responses) (Fig. 5 in manuscript). 63% of those responses were positive. Students frequently mentioned their appreciation of the highly-structured, scaffolded writing process, the general feedback given to them, and their impression that they perceived themselves as becoming better writers over the course of the semester. These self-reported comments are in line with students’ perceptions reported in the CURE survey. Given that improved writing within the discipline is one of the student learning outcomes for both FSBio 201 and the overall Allegheny FS curriculum, we find this result especially encouraging. Student comments on oral communication were less frequent (13.2% of total responses) than those for writing but still mostly positive (76%). Students frequently mentioned appreciation of practice talks and feedback from instructors. Negative comments related to written and oral communication most commonly asked for more explicit guidance.

*RSE data on peer-to-peer teaching suggest that formal training will be beneficial for teaching assistants*

Peer mentoring comments (51% negative) were mostly about the quality of feedback on writing drafts given by the undergraduate teaching assistants (Fig. 5 in manuscript). Students who made negative comments cited a lack of congruence between teaching assistant feedback on drafts and instructor feedback on the final paper, whereas positive feedback noted that teaching assistant feedback resulted in high grades on the final paper. When we choose teaching assistants for FSBio 201, we look for students with strong writing skills as evidenced by work completed for FSBio 201 and other courses. Therefore, we do not believe a lack of skill on the part of the teaching assistants has led to the negative comments. Instead, we, as instructors, likely need to spend more time mentoring our teaching assistants and finding ways to more clearly communicate our own final paper expectations to the teaching assistants, as they are providing feedback on initial paper drafts. While we do employ a standard checklist to aid in TA-provided feedback, we might address this set of student concerns by more formal training for the teaching assistants and by clarifying students’ expectations for the purpose, goals, and limitations of this form of peer mentoring.
