# Supplementary material for: Students’ Perceptions of FSBio 201, A CURE-Based Course that Scaffolds Research and Scientific Communication, Align with Learning Outcomes
Source: Integr Comp Biol. 2021 Jun 10;61(3):944–56. doi: 10.1093/icb/icab128 (PMC8490692; doi:10.1093/icb/icab128)
Supplement: icab128_Supplemental_Files [file icab128_supplemental_files.zip › icb-2021-0064-File011.docx]

**CODING GUIDELINES FOR REPORT OF STUDENT EXPERIENCE (RSE) NARRATIVES**

To minimize subjectivity, the evaluator did not code RSEs/narratives from the module that they taught.

**Semesters coded: Fall 2013 - Fall 2019**

**General information**

Each semester was coded by one evaluator using a Google form specific for the semester. If the student narrative did not comment on a particular variable, it was left blank. The same comment could be counted towards two or more categories if the contents fit into those categories. If the comment did not fit into any of the categories, the comment was still be counted towards the total number of comments.

**General comments vs. instructor specific comments:** For a given semester when two/three instructors co-taught the same FSBio 201 class, general comments were common among those instructors. General comments were coded only once.

Each variable was coded according to the definitions below:

**Positive:** Students mentioned that they enjoyed that aspect of the course or something helpful about that aspect.

**Negative**: Students mentioned that they disliked that aspect of the course or something perceived to hurt or detracted from their experience.

**Neutral:** Students mentioned the aspect of the course but did not associate it with anything positive or negative.

**Unsure:** Person scoring could not determine whether the comment fit one of the above categories (fits more than one category or cannot determine the nature of the comment).

A common sentiment found in the narratives was the indication that students needed more guidance on something. This was interpreted as negative. The negative category also includes noting a lack of something or wanting more of something. If “unsure” was chosen, the evaluators were asked to record something identifiable so that the exact comment could be re-evaluated later by the authors as a group.

**VARIABLES TO CODE**

**Contents specific to a module**

**A. Use of organisms/experimental subjects used in the module:** Comments that specifically mentioned the organisms or experimental subjects that students worked with during a specific module.

*Examples:*

Positive

“Learned a lot about yucky bugs. It made me not dislike them so much.”

"Who knew earwigs were so interesting?"

Negative

“...would have appreciated more preliminary background information on cell biology/genetics to feel more prepared to plan and administer experiments. I would add a class or half a class of basic cell bio / genetics / other relevant information.”

“I felt like I didn’t know the mechanism behind DR and at the same time I felt like it wasn’t needed to be able to do the project. I would put more focus on the mechanisms behind instead of just heart rate effects.”

Neutral

“Maybe add different drugs to be tested on the yeast.”

“..for a while I was confused about what *S. marcescens* was…”

**B. Study of different biological processes and sub-disciplines:** Comments that mentioned specific keywords in a sub-discipline (e.g. cell signaling, chemotaxis, development, biomechanics) or the name of a sub-disciplines (e.g. microbiology, cell biology, genetics, ecology).

*Examples:*

Positive

“The activities that we did explaining transmission and virulence” (comment under “aspects of this course that were most useful in helping you learn…”)

"I liked the in depth look at the chemical processes that occur in our bodies."

Negative

“Enjoy the experience even though I have no interest in ecology”

“DNA mutagenesis and how it works is a very foreign concept to me.”

Neutral

“The module was supposed to be on studying developmental effects on zebrafish.”

“Reading the paper about the dive response was also very helpful.”

**C. Experimental approaches/techniques specific to a sub-discipline**: Comments that mentioned practices we follow based on a sub-discipline and are not universal to all disciplines. The practices could be applicable to multiple sub-disciplines, but were not applicable to ALL sub-disciplines. Examples included a specific kind of data analysis (more specific than qualitative and quantitative), time-course study that required students to come in outside of lab time, and specific instrumentation or technique or assay.

*Examples:*

Positive

“Liked that I learned dilutions a little more”

“Hands on experience with electrophoresis gel and analyzing gels was a useful in helping me learn.”

Negative

“I did not like having to work in the mud.”

“Computers and equipment were kind of broken and made experimentation frustrating, not much was done to fix it.”

Neutral

“Find a way to make up own experiment utilizing barcoding (not just identification)”

“She spent a lot of time helping us w/ sterile technique, powerpoints….”

**Different facets of research universal to all sub-disciplines**

**D. Designing experiments (how to design and how to analyze/interpret data)**: Comments that mentioned aspects related to experimental designs universal to all disciplines of biology. Some cases included, but were not limited to, mentioning that they learned how to design experiments (such as including controls, biological and technical replicates, etc.) and analyze and interpret data (in some cases using statistical analyses). In this category (as opposed to category C), we considered designing experiments as a way of doing research rather than a way of engaging in a sub-discipline specific approach. When there were ambiguities, the coder made a judgment call or marked the comment as “unknown” so that the authors could decide as a group.

*Examples:*

Positive

“Figure analysis….helped”

“The aspects of this course that were most helpful were learning how to make graphs and conducting the experiments.”

Negative

“...there was minimum guidance for data analysis.”

“...but there was a lack of explanations for the lab procedures. I would spend more time practicing and analyzing lab procedures and results.”

Neutral

“More help explaining statistics and data analysis.”

“...very helpful with stats collection, which was good because that was hard.”

**E. Iterative process:** Comments that alluded to the iterative nature of research. These comments often assumed the form of a negative comment (tediousness, repetitive nature, etc.) or a positive comment (repeating the experiment, getting practice on initial experiment and repeating it to get results, etc.).

*Examples*:

Positive

“Follow up experiments to further investigate hypotheses” (comment under “aspects of this course that were most useful in helping you learn…”)

"Instructor feedback during our project was very helpful"

Negative

“The only thing I would change is to have fewer collection days for data and focus more on

analyzing and finding scientific research.”

“The initial experiment that the whole class did was not helpful because it felt more like busy work than learning.”

Neutral

No neutral comments were identified for this category.

**F. Independence to pursue one’s own ideas with guidance:** Comments mentioned something related to students’ independent projects. Scenarios included, but were not limited to, mentioning independent projects, becoming independent as time goes on, acquiring the ability to put one’s ideas into action, showing a sense of ownership or coming up with their experiment idea on their own.

*Examples:*

Positive

“...also thought creating our own experiments were helpful….”

“I also thought the fact that the class allowed us to be independent in our experiments was helpful and beneficial in allowing us to explore and learn.”

Negative

“However, I felt like our experiments were not novel and lacked significance.”

“I’m still a little confused on reading our results and explaining it in our paper. A little more direction on that would have been beneficial.”

Neutral

“possibly allow for more opportunities to experiment with alterations in the starvation process.”

“Because the experiment was already designed, it made initially learning how to write a

proper lab report /give a presentation the key focus, which was the most helpful in this case”

**G. Working with uncertainty and failure or recognizing the aspect of discover**

This category typically included at least one of the following scenarios:

- Students’ ability to recognize failure in research, either positively or negatively (equating failure with: experiment did not work as planned, contamination, hypothesis was not supported, lack of differences between experimental groups, etc.)
- Students were happy or frustrated that they could not find the answer to their research question. Some students did not realize that their research question is open-ended and we (instructors) do not know the answer to the question either. Statements that imply such a sentiment would count toward this category.
- Students’ comments on the challenges of finding relevant literature, how to interpret complex literature, how to synthesize information from literature to apply it to their findings and their frustration with literature due to the lack of one single answer.
- Students’ realization that they were the first one to know the answer to the question

*Examples:*

Positive

“...really enjoyed working with human participants and all the complexities that brings.”

“I liked that I didn’t know what role peroxidases played in the plants.”

Negative

"On its own, the research paper given to us as background for our own papers was difficult to understand w/o some kind of walkthrough/where to find relevant info within the paper"

“It was difficult finding specific articles that supported our hypotheses.”

Neutral

“Some of the material in the papers confused me and I had to put more effort into understanding

the material.”

“having a class to discuss possible experiment ideas or previous successful *Serratia* experiments may help future groups get more data.”

**H. Collaborative effort:** Comments that mentioned all aspects of group work, including learning from each other, miscommunication within the group, fairness/unfairness of working in a group, etc. This category also included, but was not limited to, student’s ability to learn from the group, student’s experience with miscommunication within the group or student thinking that it’s unfair to suffer the consequences of a dysfunctional group.

*Examples:*

Positive

“The individual research assignment had a nice balance of independent work and group work.”

“I liked being able to choose our groups…”

Negative

“I did not like performing this experiment in a group and having to rely on them making an appearance.”

“I was not always a fan of the graded work being group-based.”

Neutral

No neutral comments were identified for this category.

1. **Making connections to the broader picture:** Comments that described how their research project fits into the big picture, real world problems, application to humans or how it fits into larger biology.

*Examples:*

Positive

“I like how we study such an important part of development and make it feel simple.”

“I’m interested in water contamination and I was able to relate that to populations of various phytoplankton.”

Negative

“...there were not many larger implications of the research we conducted.”

“Didn’t see a “Big Picture”

Neutral

No neutral comments were identified for this category.

**Communication skills**

1. Writing research paper
2. Oral presentation

Comments that mentioned that students learned how to write a research paper or how to effectively communicate findings from a research project. If they used the word “communication,” we coded for both writing and oral categories since the word recognizes both skills.

*Examples for Paper Writing:*

Positive

“It was also helpful to write each section of our paper independently because we had a sufficient amount of time to revise them.”

“working bit by bit on the scientific papers was really helpful in learning how to write them. The handouts and lectures on how to improve writing for specific section of the paper were very helpful”

Negative

"...was just teaching us to copy something vs how to write on our own"

“Many different professors want/ask for different things on the report. Inconsistency is

slightly confusing.”

Neutral

"I think it would be helpful for students to turn in their final draft with the professor's edits one last time with their final corrections. That way students can perfect their writing skills"

“I would have liked more guidance on how to write the paper; guidelines for the sections or pros and cons”

*Examples for Oral Presentations:*

Positive

“Quick & helpful feedback on drafts and practice presentations → helpful in editing & fine-tuning final products”

“...direct feedback after presentations,...”

Negative

“Presentations were least helpful because we didn’t receive adequate feedback to better ourselves.”

“I feel like we could have focused more on how to structure a good talk…”

Neutral

“I only thought that maybe having the informal presentations graded would be necessary.”

“I think that it would really be helpful to go over our presentations each week with us,

specifically go from slide to slide for each graph and discuss formatting and wording and

the topic in general. Everyone learns from each graph in this way (this is what was done

in Prof. X’s module and it was the most helpful part from all of FSBio).”

**Others**

**Students’ perception of peer-to-peer teaching from TAs:** Comments that mentioned TAs’s assistance in the context of the research process (planning or executing experiments, data analysis, technical assistance, etc.) or communication (writing paper and oral presentation).

*Examples:*

Positive

“The responses from the peer leaders were wonderful because they provided a lot of helpful feedback for my first scientific paper.”

“Having a TA and professor comment on my papers were very helpful”

Negative

"least helpful was getting TA comments that differed from won writing style and also what Professor X would say"

“The TAs should have a great understanding of what that professor is looking for when grading drafts, and it was evident in one module that the understanding between professor and TAs was not good”

Neutral

“More peer editing!!”

“I feel that not only TAs should make corrections o r take a look at our papers because

sometimes their perspective is different than the professors.”

**Students’ desire to learn more about the topic or further extend the research:** If the student mentioned that they would like to know more about the topic or pursue research in a particular sub-discipline, the comment counted towards this category. For this category, “yes,” “no” and “unsure” were the options.

**General Comments:** If the student mentioned their general likes or dislikes about the module without getting into a specific aspect of the module, the comment counted towards this category.

**Other comments:** If the comment did not count towards any of the categories above and was considered noteworthy, the authors recorded the comment.
