# Supplementary material for: Students’ Perceptions of FSBio 201, A CURE-Based Course that Scaffolds Research and Scientific Communication, Align with Learning Outcomes
Source: Integr Comp Biol. 2021 Jun 10;61(3):944–56. doi: 10.1093/icb/icab128 (PMC8490692; doi:10.1093/icb/icab128)
Supplement: icab128_Supplemental_Files [file icab128_supplemental_files.zip › icb-2021-0064-File012.docx]

**Supplemental data for “model organism”, “biological process”, “experimental approach”, and “experimental design”.**

Model organism:

As we scored comments for this category as generally positive, negative, or neutral, we also kept track of which specific organisms were mentioned. The table below contains our raw data with organisms sorted roughly taxonomically. The graph shows data aggregated by Kingdom, with “other” containing data for UZOS cells and cancer cells.

| Organism | Positive | Negative | Neutral | *Sum* |
| --- | --- | --- | --- | --- |
| Bacteria | 4 | 0 | 0 | *4* |
| *Serratia (marcescens)* | 5 | 2 | 2 | *9* |
| “Dicty”*/D. discoideum*/slime mold | 10 | 7 | 2 | *19* |
| Amoeba | 1 | 0 | 0 | *1* |
| Algae/Phytoplankton | 12 | 4 | 5 | *21* |
| Plants | 10 | 2 | 0 | *12* |
| Plant seeds | 1 | 0 | 0 | *1* |
| Goldenrod | 4 | 1 | 0 | *5* |
| Goldenrod galls | 5 | 1 | 0 | *6* |
| *Batrachochytrium dendrobatidis* | 3 | 0 | 0 | *3* |
| Yeast | 2 | 0 | 1 | *3* |
| Animals | 1 | 0 | 0 | *1* |
| Fruit flies | 2 | 1 | 1 | *4* |
| Crayfish | 2 | 0 | 0 | *2* |
| Earwig | 8 | 3 | 1 | *12* |
| Insects/Bugs | 2 | 1 | 1 | *4* |
| Fish | 0 | 2 | 1 | *3* |
| Zebrafish | 2 | 2 | 7 | *11* |
| Shark teeth/Sharks | 12 | 2 | 3 | *17* |
| Amphibians | 1 | 0 | 0 | *1* |
| Tadpoles | 2 | 0 | 0 | *2* |
| Toads | 1 | 0 | 0 | *1* |
| Salamanders | 20 | 3 | 3 | *26* |
| Humans | 15 | 1 | 1 | *17* |
| UZOS/UZOS cells | 3 | 0 | 0 | *3* |
| Cancer cells/cancer | 3 | 0 | 0 | *3* |


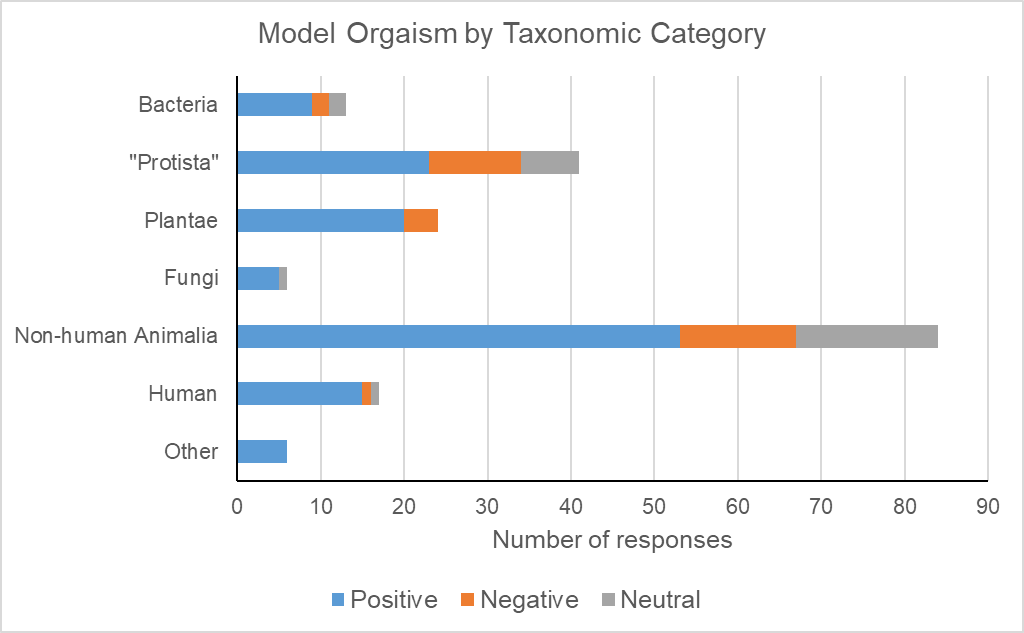


Biological process

As we scored comments for this category as generally positive, negative, or neutral, we also kept track of which specific biological processes were mentioned. The tables below contain our raw data divided by scope (molecular/cellular, organismal/physiological, populations/ecosystems). The graph shows data aggregated by these three categories. One mention of “living things” (positive), is not represented below.

| Molecular/cellular processes | Positive | Negative | Neutral | *Sum* |
| --- | --- | --- | --- | --- |
| Peroxidases | 7 | 12 | 5 | *24* |
| Chemotaxis | 10 | 2 | 4 | *16* |
| Cell biology | 4 | 1 | 0 | *5* |
| Aggregation | 1 | 1 | 1 | *3* |
| Genetics | 1 | 1 | 0 | *2* |
| Pathways | 2 | 0 | 0 | *2* |
| Cells reacting | 1 | 0 | 0 | *1* |
| cAMP regulation | 1 | 0 | 0 | *1* |
| Chemiosmosis | 1 | 0 | 0 | *1* |
| Chemical attractants | 1 | 0 | 0 | *1* |
| Developmental effects | 0 | 0 | 1 | *1* |
| DNA mutagenesis | 0 | 1 | 0 | *1* |
| DNA repair | 1 | 0 | 0 | *1* |
| Fly phenotypes | 1 | 0 | 0 | *1* |
| Molecular biology | 1 | 0 | 0 | *1* |
| SNP genetics | 0 | 1 | 0 | *1* |
| Starvation process | 0 | 0 | 1 | *1* |

| Organismal/physiological processes | Positive | Negative | Neutral | *Sum* |
| --- | --- | --- | --- | --- |
| Dive response | 13 | 8 | 2 | *23* |
| Microbiology | 7 | 1 | 1 | *9* |
| Development/developmental biology | 4 | 1 | 0 | *5* |
| Biomechanics/biophysics | 1 | 1 | 2 | *4* |
| Physiology | 3 | 0 | 0 | *3* |
| Assessing metabolic activity | 0 | 1 | 0 | *1* |
| Circulatory system | 1 | 0 | 0 | *1* |
| Crayfish anatomy | 1 | 0 | 0 | *1* |
| Embryology | 0 | 0 | 1 | *1* |
| Heart | 1 | 0 | 0 | *1* |
| Heart response to drug | 1 | 0 | 0 | *1* |
| Nervous system | 1 | 0 | 0 | *1* |
| Neuroscience | 1 | 0 | 0 | *1* |
| Plant defenses | 1 | 0 | 0 | 1 |
| Shark feeding | 1 | 0 | 0 | *1* |
| Sympathetic/parasympathetic | 0 | 0 | 1 | *1* |
| Tooth puncture | 1 | 0 | 0 | *1* |

| Population/ecosystem processes | Positive | Negative | Neutral | *Sum* |
| --- | --- | --- | --- | --- |
| Behavior | 5 | 0 | 0 | *5* |
| Disease/transmission | 3 | 0 | 0 | *3* |
| Ecology | 1 | 1 | 0 | *2* |
| Mating/sex | 2 | 0 | 0 | *2* |
| Species interactions | 2 | 0 | 0 | *2* |
| Disease ecology | 1 | 0 | 0 | *1* |
| Disease simulation | 1 | 0 | 0 | *1* |
| Maternal care | 1 | 0 | 0 | *1* |
| Physiological ecology | 1 | 0 | 0 | *1* |
| Sexual selection | 1 | 0 | 0 | *1* |
| Virulence | 1 | 0 | 0 | *1* |


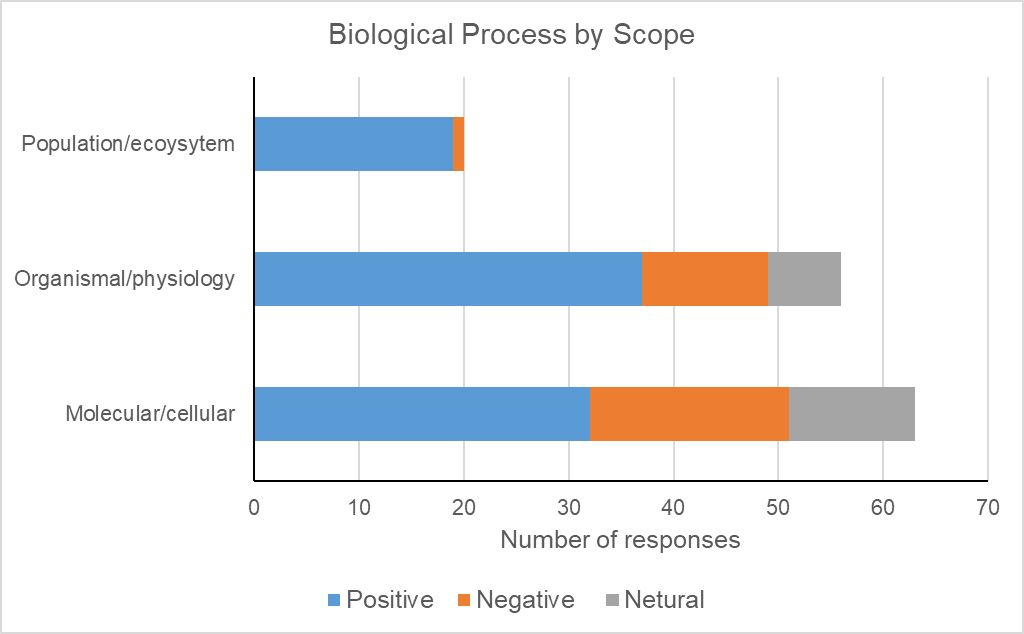


Experimental design

As we scored comments for this category as generally positive, negative, or neutral, we also kept track of which specific experimental design elements were mentioned. The table below shows our raw data.

| Experimental design element | Positive | Negative | Neutral | *Sum* |
| --- | --- | --- | --- | --- |
| Statistics | 20 | 32 | 5 | *57* |
| Data analysis | 12 | 11 | 1 | *24* |
| Experimental design (general) | 11 | 4 | 0 | *15* |
| Graphing/making figures | 7 | 8 | 0 | *15* |
| Data interpretation | 5 | 3 | 0 | *8* |
| Figure analysis | 5 | 0 | 0 | *5* |
| General research/experience | 5 | 0 | 0 | *5* |
| Hands on/interactive | 4 | 0 | 0 | *4* |
| Model/experiment simplicity | 3 | 0 | 0 | *3* |
| Data | 0 | 2 | 0 | *2* |
| Choosing hypotheses | 1 | 1 | 0 | *2* |
| Excel | 1 | 1 | 0 | *2* |
| General lab techniques | 2 | 0 | 0 | *2* |
| Interpreting figures | 1 | 1 | 0 | *2* |
| Computer processing | 0 | 1 | 0 | *1* |
| Background research | 1 | 0 | 0 | *1* |
| Computer programs (general) | 0 | 1 | 0 | *1* |
| Computer systems | 1 | 0 | 0 | *1* |
| Data collection | 0 | 1 | 0 | *1* |
| Having a plan | 1 | 0 | 0 | *1* |
| Learning to plan | 1 | 0 | 0 | *1* |
| Numerical data | 0 | 1 | 0 | *1* |
| Picking samples & organism identification | 0 | 1 | 0 | *1* |
| Pilot experiment | 1 | 0 | 0 | *1* |
| Thoroughness | 1 | 0 | 0 | *1* |
| Using a model paper | 1 | 0 | 0 | *1* |

Experimental approach

As we scored comments for this category as generally positive, negative, or neutral, we also kept track of which specific experimental approaches were mentioned. The table below shows our raw data.

| Experimental Approach | Positive | Negative | Neutral | *Sum* |
| --- | --- | --- | --- | --- |
| Outdoors/fieldwork | 52 | 4 | 0 | *56* |
| Microscopes | 13 | 10 | 1 | *24* |
| Coming in outside of class | 1 | 13 | 0 | *14* |
| DNA barcoding | 11 | 1 | 2 | *14* |
| Waiting/time required | 1 | 10 | 0 | *11* |
| Live animals/organisms | 10 | 0 | 0 | *10* |
| Sterile technique | 8 | 0 | 1 | *9* |
| Counting cells/organisms | 4 | 4 | 0 | *8* |
| Electrophoresis | 7 | 0 | 0 | *7* |
| Dissection | 6 | 0 | 0 | *6* |
| JMP | 3 | 3 | 0 | *6* |
| Dilutions/serial dilutions | 3 | 1 | 0 | *4* |
| Tools | 2 | 2 | 0 | *4* |
| Micropipette | 2 | 1 | 0 | *3* |
| Drugs | 2 | 0 | 0 | *2* |
| Finding human subjects | 1 | 1 | 0 | *2* |
| Gels | 2 | 0 | 0 | *2* |
| Making DNA database | 2 | 0 | 0 | *2* |
| Observing/scoring plates | 0 | 0 | 2 | *2* |
| qPCR | 2 | 0 | 0 | *2* |
| Wells | 1 | 1 | 0 | *2* |
| CSI lab | 1 | 0 | 0 | *1* |
| Equipment | 1 | 0 | 0 | *1* |
| Exploratory | 1 | 0 | 0 | *1* |
| Genetics work | 1 | 0 | 0 | *1* |
| Software | 0 | 1 | 0 | *1* |
| Working with cells | 1 | 0 | 0 | *1* |
| Computer program | 0 | 1 | 0 | *1* |
| Epidemiology | 1 | 0 | 0 | *1* |
| Epi-info | 1 | 0 | 0 | *1* |
| Estimating | 0 | 1 | 0 | *1* |
| Excel | 1 | 0 | 0 | *1* |
| Hands-on | 1 | 0 | 0 | *1* |
| Isoenzymes | 1 | 0 | 0 | *1* |
| Isolating DNA | 0 | 1 | 0 | *1* |
| Observing cells | 1 | 0 | 0 | *1* |
| PCR | 1 | 0 | 0 | *1* |
| Pipetting | 1 | 0 | 0 | *1* |
| Planting seeds | 1 | 0 | 0 | *1* |
| Standards for curve | 0 | 1 | 0 | *1* |
| Taxonomy | 1 | 0 | 0 | *1* |
| Tracker | 1 | 0 | 0 | *1* |
| UV crosslinker | 0 | 1 | 0 | *1* |
